# Tricarbonyl­bis(tricyclo­hexyl­phosphine-κ*P*)ruthenium(0) toluene solvate

**DOI:** 10.1107/S1600536808034211

**Published:** 2008-10-22

**Authors:** Andreas Nader, Helmar Görls, Wolfgang Imhof

**Affiliations:** a Institut für Anorganische und Analytische Chemie, Friedrich-Schiller-Universität, August-Bebel-Strasse 2, 07743 Jena, Germany

## Abstract

The title compound, [Ru(C_18_H_33_P)_2_(CO)_3_]·C_7_H_8_, shows a distorted trigonal-bipyramdial coordination around the central Ru atom, with the two phosphine ligands occupying the axial positions. Two toluene mol­ecules per asymmetric unit with site-occupation factors of 0.5 are observed. One of them forces two of the CO ligands to enclose a wider C—Ru—C bond angle [127.5 (3)°] than in the solvent-free crystal structure of [Ru(PCy_3_)_2_(CO)_3_] (Cy is cyclo­hexyl).

## Related literature

For background, see: Berger & Imhof (1999 ▶), Dönnecke & Imhof (2003 ▶), Chaudret & Poilblanc (1985 ▶), Song & Trogler (1992 ▶). For the solvent-free structure, see: Dunne *et al.* (2004 ▶). 
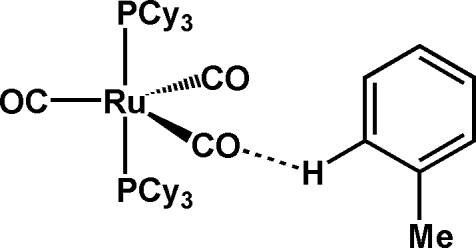


## Experimental

### 

#### Crystal data

[Ru(C_18_H_33_P)_2_(CO)_3_]·C_7_H_8_
*M* *_r_* = 838.06
Triclinic, 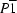

*a* = 12.4367 (6) Å
*b* = 12.4980 (4) Å
*c* = 16.2970 (7) Å
α = 92.685 (2)°
β = 103.594 (2)°
γ = 103.500 (2)°
*V* = 2380.2 (2) Å^3^
*Z* = 2
Mo *K*α radiation
μ = 0.43 mm^−1^
*T* = 183 (2) K
0.08 × 0.06 × 0.05 mm

#### Data collection

Nonius KappaCCD diffractometer
Absorption correction: none
16605 measured reflections
10678 independent reflections
7032 reflections with *I* > 2σ(*I*)
*R*_int_ = 0.051

#### Refinement

*R*[*F* ^2^ > 2σ(*F* ^2^)] = 0.082
*wR*(*F* ^2^) = 0.243
*S* = 1.05
10678 reflections
438 parameters
H-atom parameters constrained
Δρ_max_ = 3.45 e Å^−3^
Δρ_min_ = −0.64 e Å^−3^

### 

Data collection: *COLLECT* (Nonius, 1998 ▶); cell refinement: *DENZO* (Otwinowski & Minor, 1997 ▶); data reduction: *DENZO*; program(s) used to solve structure: *SHELXS97* (Sheldrick, 2008 ▶); program(s) used to refine structure: *SHELXL97* (Sheldrick, 2008 ▶); molecular graphics: *XP* (Siemens, 1990 ▶); software used to prepare material for publication: *XP*.

## Supplementary Material

Crystal structure: contains datablocks I, global. DOI: 10.1107/S1600536808034211/hb2822sup1.cif

Structure factors: contains datablocks I. DOI: 10.1107/S1600536808034211/hb2822Isup2.hkl

Additional supplementary materials:  crystallographic information; 3D view; checkCIF report

## Figures and Tables

**Table:** 

Ru1—C1	1.903 (6)
Ru1—C3	1.915 (6)
Ru1—C2	1.919 (7)
Ru1—P1	2.3777 (15)
Ru1—P2	2.3780 (15)

**Table:** 

P1—Ru1—P2	176.22 (5)

**Fig. 1.**
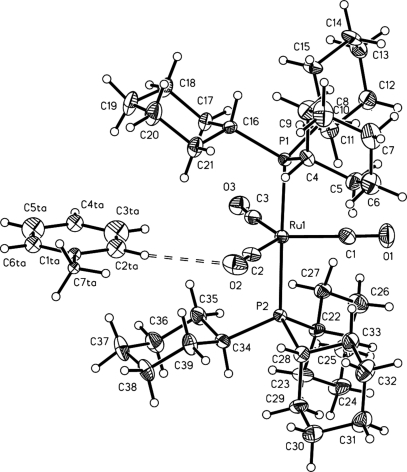
Molecular structure of (I) with isplacement ellipsoids for the non-hydrogen atoms drawn at the 50% probability level.

## supplementary crystallographic information

### Comment

In the course of a study whether Ru(II) complexes might act as suitable pre-catalysts in the reaction of α,β-unsaturated imines with carbon monoxide and ethylene to produce chiral γ-lactams, which is originally catalyzed by Ru(0) compounds (Berger & Imhof, 1999; Dönnecke & Imhof, 2003), we intended to use the non-classical Ru(II) complex [Ru(PCy_3_)_2_(H_2_)_2_(H)_2_] (Chaudret & Poilblanc, 1985) as the precatalyst. After cooling down the autoclave a white precipitate of the title compound was collected. This means that obviously carbon monoxide has replaced all dihydrogen and hydride ligands and the ruthenium center has been reduced from Ru(II) to Ru(0).

The molecular structure of the title compound has been published before as a solvent free crystal structure (*P*2_1_/*n*, Dunne *et al.*, 2004) with one disordered cyclohexyl ring. The synthesis at that time followed a literature procedure that used RuCl_3_.H_2_O, KOH, PCy_3_ and formaldehyde as reducing agent and source of carbon monoxide (Song & Trogler, 1992). The bond lengths in both structures are identical within systematic errors. Nevertheless, the C—Ru—C bond angles in the Ru(CO)_3_ plane are significantly different with 119.74 (9)°, 116.77 (9)° and 123.49 (9)° in the case of the structure reported by Dunne *et al.* whereas the corresponding angles in (I) measure to 109.3 (3)°, 123.2 (2)° and 127.5 (3)°. This difference is most probably caused by one of the disordered toluene solvent molecules being situated between two cyclohexyl rings of the phosphine ligands therefore leading to the highest observed bond angle of 127.6 (3)° (C2—Ru1—C3, Figure 1). In addition, one of the aromatic hydrogen atoms shows a weak C—H···O interaction towards one of the carbon monoxide ligands (H2TA···O2 = 2.09 (2) Å).

### Experimental

In an attempt to catalytically react methyl-(3-phenylallylidene)amine with carbon monoxide and ethylene, 1 mmol of the imine together with 0.03 mmol (20 mg) [Ru(PCy_3_)_2_(H_2_)_2_(H)_2_] were dissolved in 4 ml toluene and were heated to 413 K for 17 h in an autoclave pressurized with 8 bar ethylene and 12 bar carbon monoxide. After cooling down the autoclave a white precipitate had formed which was collected and recrystallized from toluene to give colourless prisms of (I) (yield based on Ru: 48%).

### Refinement

The two solvent toluene molecules have been refined isotropically with sof's of 0.5 and have been constrained to be regular hexagons by AFIX 66 instructions in SHELXL. Hydrogen atoms were placed in idealized positions and refined as riding with U_iso_(H) = 1.2 *U*_eq_(C).

### Crystal data

**Table:**

[Ru(C_18_H_33_P)_2_(CO)_3_]·C_7_H_8_	*Z* = 2
*M**_r_* = 838.06	*F*(000) = 896
Triclinic, *P*1	*D*_x_ = 1.169 Mg m^−^^3^
Hall symbol: -P 1	Mo *K*α radiation, λ = 0.71073 Å
*a* = 12.4367 (6) Å	Cell parameters from 16612 reflections
*b* = 12.4980 (4) Å	θ = 2.2–27.5°
*c* = 16.2970 (7) Å	µ = 0.43 mm^−^^1^
α = 92.685 (2)°	*T* = 183 K
β = 103.594 (2)°	Prism, colourless
γ = 103.500 (2)°	0.08 × 0.06 × 0.05 mm
*V* = 2380.2 (2) Å^3^	

### Data collection

**Table:**

Nonius KappaCCD diffractometer	7032 reflections with *I* > 2σ(*I*)
Radiation source: fine-focus sealed tube	*R*_int_ = 0.051
graphite	θ_max_ = 27.5°, θ_min_ = 2.3°
φ and ω scans	*h* = −16→15
16605 measured reflections	*k* = −16→16
10678 independent reflections	*l* = −21→21

### Refinement

**Table:**

Refinement on *F*^2^	Primary atom site location: structure-invariant direct methods
Least-squares matrix: full	Secondary atom site location: difference Fourier map
*R*[*F*^2^ > 2σ(*F*^2^)] = 0.082	Hydrogen site location: inferred from neighbouring sites
*wR*(*F*^2^) = 0.243	H-atom parameters constrained
*S* = 1.05	*w* = 1/[σ^2^(*F*_o_^2^) + (0.123*P*)^2^ + 7.5629*P*] where *P* = (*F*_o_^2^ + 2*F*_c_^2^)/3
10678 reflections	(Δ/σ)_max_ < 0.001
438 parameters	Δρ_max_ = 3.45 e Å^−^^3^
0 restraints	Δρ_min_ = −0.64 e Å^−^^3^

### Special details

**Table:**

Geometry. All e.s.d.'s (except the e.s.d. in the dihedral angle between two l.s. planes) are estimated using the full covariance matrix. The cell e.s.d.'s are taken into account individually in the estimation of e.s.d.'s in distances, angles and torsion angles; correlations between e.s.d.'s in cell parameters are only used when they are defined by crystal symmetry. An approximate (isotropic) treatment of cell e.s.d.'s is used for estimating e.s.d.'s involving l.s. planes.
Refinement. Refinement of *F*^2^ against ALL reflections. The weighted *R*-factor *wR* and goodness of fit *S* are based on *F*^2^, conventional *R*-factors *R* are based on *F*, with *F* set to zero for negative *F*^2^. The threshold expression of *F*^2^ > σ(*F*^2^) is used only for calculating *R*-factors(gt) *etc*. and is not relevant to the choice of reflections for refinement. *R*-factors based on *F*^2^ are statistically about twice as large as those based on *F*, and *R*- factors based on ALL data will be even larger.

### Fractional atomic coordinates and isotropic or equivalent isotropic displacement parameters (Å^2^)

**Table:**

	*x*	*y*	*z*	*U*_iso_*/*U*_eq_	Occ. (<1)
Ru1	0.13721 (4)	0.23221 (4)	0.23002 (3)	0.01644 (16)	
P1	−0.01593 (12)	0.08021 (12)	0.23565 (9)	0.0150 (3)	
P2	0.29082 (12)	0.38833 (12)	0.23373 (9)	0.0155 (3)	
O1	−0.0288 (5)	0.3806 (4)	0.1972 (3)	0.0409 (13)	
O2	0.2609 (5)	0.1948 (4)	0.4093 (3)	0.0409 (13)	
O3	0.1465 (4)	0.1397 (4)	0.0545 (3)	0.0308 (11)	
C1	0.0340 (5)	0.3249 (5)	0.2116 (4)	0.0233 (13)	
C2	0.2164 (5)	0.2079 (5)	0.3409 (4)	0.0233 (13)	
C3	0.1466 (5)	0.1712 (5)	0.1227 (4)	0.0219 (13)	
C4	−0.0556 (5)	0.0908 (5)	0.3394 (4)	0.0200 (12)	
H4A	0.0151	0.0920	0.3843	0.024*	
C5	−0.0852 (6)	0.2003 (5)	0.3570 (4)	0.0263 (14)	
H5A	−0.1539	0.2045	0.3132	0.032*	
H5B	−0.0212	0.2624	0.3530	0.032*	
C6	−0.1078 (7)	0.2126 (6)	0.4456 (4)	0.0357 (17)	
H6A	−0.0364	0.2176	0.4897	0.043*	
H6B	−0.1317	0.2819	0.4530	0.043*	
C7	−0.1996 (7)	0.1160 (6)	0.4570 (5)	0.0383 (18)	
H7A	−0.2729	0.1147	0.4163	0.046*	
H7B	−0.2099	0.1245	0.5152	0.046*	
C8	−0.1670 (7)	0.0065 (7)	0.4425 (5)	0.0410 (19)	
H8A	−0.0962	0.0058	0.4854	0.049*	
H8B	−0.2287	−0.0563	0.4489	0.049*	
C9	−0.1485 (6)	−0.0064 (6)	0.3538 (4)	0.0320 (16)	
H9A	−0.2212	−0.0110	0.3111	0.038*	
H9B	−0.1261	−0.0763	0.3456	0.038*	
C10	−0.1535 (5)	0.0670 (5)	0.1531 (4)	0.0205 (12)	
H10A	−0.1949	0.1151	0.1774	0.025*	
C11	−0.1395 (6)	0.1110 (5)	0.0687 (4)	0.0230 (13)	
H11A	−0.0894	0.1872	0.0799	0.028*	
H11B	−0.1030	0.0637	0.0396	0.028*	
C12	−0.2566 (6)	0.1113 (6)	0.0115 (4)	0.0292 (15)	
H12A	−0.2891	0.1642	0.0388	0.035*	
H12B	−0.2463	0.1374	−0.0433	0.035*	
C13	−0.3400 (6)	−0.0013 (6)	−0.0056 (4)	0.0316 (15)	
H13A	−0.4155	0.0045	−0.0392	0.038*	
H13B	−0.3127	−0.0527	−0.0390	0.038*	
C14	−0.3518 (5)	−0.0462 (6)	0.0773 (4)	0.0301 (15)	
H14A	−0.4032	−0.1217	0.0654	0.036*	
H14B	−0.3869	0.0012	0.1077	0.036*	
C15	−0.2352 (5)	−0.0500 (5)	0.1343 (4)	0.0263 (14)	
H15A	−0.2017	−0.1005	0.1055	0.032*	
H15B	−0.2456	−0.0788	0.1882	0.032*	
C16	0.0086 (5)	−0.0600 (4)	0.2290 (4)	0.0175 (12)	
H16A	−0.0650	−0.1132	0.2294	0.021*	
C17	0.0376 (6)	−0.0909 (5)	0.1457 (4)	0.0226 (13)	
H17A	0.1146	−0.0460	0.1457	0.027*	
H17B	−0.0183	−0.0742	0.0970	0.027*	
C18	0.0350 (6)	−0.2138 (5)	0.1360 (4)	0.0295 (15)	
H18A	−0.0433	−0.2587	0.1322	0.035*	
H18B	0.0555	−0.2319	0.0828	0.035*	
C19	0.1188 (7)	−0.2425 (6)	0.2115 (5)	0.0390 (18)	
H19A	0.1092	−0.3236	0.2072	0.047*	
H19B	0.1979	−0.2073	0.2093	0.047*	
C20	0.1012 (7)	−0.2042 (6)	0.2962 (5)	0.0384 (18)	
H20A	0.1632	−0.2161	0.3427	0.046*	
H20B	0.0278	−0.2497	0.3032	0.046*	
C21	0.0998 (6)	−0.0801 (5)	0.3039 (4)	0.0292 (15)	
H21A	0.0826	−0.0602	0.3580	0.035*	
H21B	0.1758	−0.0333	0.3035	0.035*	
C22	0.2621 (5)	0.4827 (5)	0.1498 (4)	0.0196 (12)	
H22A	0.2237	0.5349	0.1728	0.024*	
C23	0.3703 (6)	0.5562 (5)	0.1305 (4)	0.0287 (15)	
H23A	0.4093	0.5091	0.1038	0.034*	
H23B	0.4238	0.5952	0.1842	0.034*	
C24	0.3370 (6)	0.6416 (5)	0.0703 (4)	0.0334 (16)	
H24A	0.3012	0.6907	0.0983	0.040*	
H24B	0.4067	0.6879	0.0583	0.040*	
C25	0.2547 (6)	0.5848 (6)	−0.0122 (4)	0.0321 (16)	
H25A	0.2926	0.5407	−0.0427	0.039*	
H25B	0.2323	0.6412	−0.0487	0.039*	
C26	0.1487 (6)	0.5097 (6)	0.0043 (5)	0.0330 (16)	
H26A	0.0982	0.4700	−0.0502	0.040*	
H26B	0.1063	0.5552	0.0289	0.040*	
C27	0.1789 (5)	0.4252 (5)	0.0652 (4)	0.0234 (13)	
H27A	0.2142	0.3747	0.0382	0.028*	
H27B	0.1081	0.3803	0.0765	0.028*	
C28	0.3294 (5)	0.4788 (5)	0.3363 (4)	0.0203 (12)	
H28A	0.3492	0.4305	0.3818	0.024*	
C29	0.4342 (6)	0.5777 (5)	0.3506 (4)	0.0299 (15)	
H29A	0.5005	0.5513	0.3425	0.036*	
H29B	0.4186	0.6299	0.3082	0.036*	
C30	0.4633 (6)	0.6376 (6)	0.4396 (5)	0.0376 (18)	
H30A	0.5303	0.7015	0.4466	0.045*	
H30B	0.4839	0.5869	0.4820	0.045*	
C31	0.3628 (7)	0.6782 (6)	0.4553 (5)	0.0380 (18)	
H31A	0.3825	0.7141	0.5142	0.046*	
H31B	0.3460	0.7336	0.4160	0.046*	
C32	0.2599 (7)	0.5832 (6)	0.4417 (5)	0.0362 (17)	
H32A	0.1942	0.6113	0.4489	0.043*	
H32B	0.2744	0.5323	0.4854	0.043*	
C33	0.2292 (6)	0.5190 (6)	0.3534 (4)	0.0288 (15)	
H33A	0.2057	0.5674	0.3098	0.035*	
H33B	0.1635	0.4546	0.3489	0.035*	
C34	0.4301 (5)	0.3614 (5)	0.2278 (4)	0.0203 (12)	
H34A	0.4832	0.4348	0.2271	0.024*	
C35	0.4209 (5)	0.2904 (5)	0.1459 (4)	0.0259 (14)	
H35A	0.3834	0.3229	0.0962	0.031*	
H35B	0.3736	0.2148	0.1460	0.031*	
C36	0.5405 (6)	0.2846 (6)	0.1388 (4)	0.0337 (16)	
H36A	0.5335	0.2369	0.0866	0.040*	
H36B	0.5857	0.3596	0.1345	0.040*	
C37	0.6021 (6)	0.2384 (7)	0.2154 (5)	0.0399 (18)	
H37A	0.6815	0.2428	0.2120	0.048*	
H37B	0.5633	0.1595	0.2146	0.048*	
C38	0.6047 (6)	0.3024 (6)	0.2994 (4)	0.0308 (15)	
H38A	0.6383	0.2651	0.3476	0.037*	
H38B	0.6542	0.3781	0.3043	0.037*	
C39	0.4861 (6)	0.3095 (6)	0.3051 (4)	0.0274 (14)	
H39A	0.4920	0.3552	0.3580	0.033*	
H39B	0.4384	0.2346	0.3065	0.033*	
C1TA	0.4931 (7)	−0.0455 (6)	0.3912 (4)	0.014 (2)*	0.50
C2TA	0.4173 (7)	0.0083 (7)	0.3442 (5)	0.032 (3)*	0.50
H2TA	0.3851	0.0562	0.3723	0.038*	0.50
C3TA	0.3887 (8)	−0.0082 (8)	0.2560 (5)	0.053 (4)*	0.50
H3TA	0.3369	0.0285	0.2238	0.063*	0.50
C4TA	0.4358 (8)	−0.0784 (8)	0.2148 (4)	0.025 (3)*	0.50
H4TA	0.4162	−0.0896	0.1546	0.030*	0.50
C5TA	0.5116 (8)	−0.1322 (7)	0.2619 (5)	0.045 (4)*	0.50
H5TA	0.5438	−0.1801	0.2338	0.054*	0.50
C6TA	0.5402 (7)	−0.1157 (7)	0.3501 (5)	0.024 (3)*	0.50
H6TA	0.5920	−0.1524	0.3823	0.028*	0.50
C7TA	0.5189 (9)	−0.0327 (9)	0.4744 (6)	0.012 (2)*	0.50
H7TA	0.4794	0.0192	0.4935	0.017*	0.50
H7TB	0.6017	−0.0034	0.4964	0.017*	0.50
H7TC	0.4949	−0.1043	0.4954	0.017*	0.50
C1TB	0.2465 (7)	0.3731 (7)	−0.2461 (4)	0.030 (3)*	0.50
C2TB	0.1736 (7)	0.4430 (7)	−0.2509 (4)	0.022 (2)*	0.50
H2TB	0.1634	0.4756	−0.2004	0.026*	0.50
C3TB	0.1159 (7)	0.4651 (7)	−0.3297 (5)	0.032 (3)*	0.50
H3TB	0.0661	0.5129	−0.3330	0.038*	0.50
C4TB	0.1309 (8)	0.4173 (8)	−0.4035 (4)	0.047 (4)*	0.50
H4TB	0.0914	0.4324	−0.4574	0.056*	0.50
C5TB	0.2038 (8)	0.3474 (8)	−0.3987 (4)	0.031 (3)*	0.50
H5TB	0.2141	0.3148	−0.4492	0.038*	0.50
C6TB	0.2615 (8)	0.3253 (7)	−0.3199 (5)	0.041 (4)*	0.50
H6TB	0.3113	0.2776	−0.3166	0.049*	0.50
C7TB	0.3013 (13)	0.3540 (12)	−0.1693 (9)	0.035 (3)*	0.50
H7TD	0.2796	0.3945	−0.1254	0.053*	0.50
H7TE	0.3840	0.3791	−0.1624	0.053*	0.50
H7TF	0.2806	0.2746	−0.1638	0.053*	0.50

### Atomic displacement parameters (Å^2^)

**Table:**

	*U*^11^	*U*^22^	*U*^33^	*U*^12^	*U*^13^	*U*^23^
Ru1	0.0155 (3)	0.0161 (2)	0.0170 (3)	0.00294 (18)	0.00400 (18)	0.00047 (17)
P1	0.0145 (8)	0.0157 (7)	0.0146 (7)	0.0036 (6)	0.0033 (6)	0.0013 (6)
P2	0.0138 (7)	0.0156 (7)	0.0160 (7)	0.0030 (6)	0.0026 (6)	0.0011 (6)
O1	0.039 (3)	0.042 (3)	0.047 (3)	0.025 (3)	0.006 (3)	0.006 (2)
O2	0.036 (3)	0.050 (3)	0.026 (3)	0.003 (2)	−0.005 (2)	0.008 (2)
O3	0.035 (3)	0.033 (3)	0.024 (2)	0.005 (2)	0.011 (2)	−0.005 (2)
C1	0.021 (3)	0.028 (3)	0.021 (3)	0.005 (3)	0.006 (3)	0.003 (3)
C2	0.019 (3)	0.024 (3)	0.023 (3)	−0.001 (3)	0.003 (3)	0.001 (3)
C3	0.020 (3)	0.021 (3)	0.023 (3)	0.002 (2)	0.003 (3)	0.003 (3)
C4	0.016 (3)	0.028 (3)	0.017 (3)	0.007 (2)	0.004 (2)	0.003 (2)
C5	0.031 (4)	0.021 (3)	0.031 (4)	0.006 (3)	0.015 (3)	0.006 (3)
C6	0.039 (4)	0.043 (4)	0.029 (4)	0.012 (3)	0.017 (3)	−0.007 (3)
C7	0.045 (5)	0.056 (5)	0.024 (4)	0.022 (4)	0.017 (3)	0.009 (3)
C8	0.049 (5)	0.051 (5)	0.039 (4)	0.022 (4)	0.029 (4)	0.022 (4)
C9	0.037 (4)	0.031 (4)	0.033 (4)	0.006 (3)	0.021 (3)	0.008 (3)
C10	0.019 (3)	0.019 (3)	0.023 (3)	0.005 (2)	0.005 (2)	0.002 (2)
C11	0.028 (4)	0.022 (3)	0.016 (3)	0.005 (3)	0.001 (3)	0.006 (2)
C12	0.030 (4)	0.032 (3)	0.023 (3)	0.011 (3)	−0.001 (3)	0.002 (3)
C13	0.022 (4)	0.037 (4)	0.033 (4)	0.009 (3)	0.001 (3)	−0.001 (3)
C14	0.015 (3)	0.037 (4)	0.030 (4)	−0.002 (3)	0.001 (3)	−0.002 (3)
C15	0.018 (3)	0.026 (3)	0.028 (3)	−0.003 (3)	0.001 (3)	0.001 (3)
C16	0.017 (3)	0.014 (3)	0.019 (3)	0.002 (2)	0.001 (2)	0.001 (2)
C17	0.027 (3)	0.021 (3)	0.023 (3)	0.012 (3)	0.006 (3)	0.003 (2)
C18	0.034 (4)	0.022 (3)	0.037 (4)	0.013 (3)	0.011 (3)	0.001 (3)
C19	0.048 (5)	0.030 (4)	0.047 (5)	0.025 (3)	0.013 (4)	0.002 (3)
C20	0.048 (5)	0.035 (4)	0.034 (4)	0.024 (4)	0.002 (3)	0.005 (3)
C21	0.035 (4)	0.027 (3)	0.027 (3)	0.016 (3)	0.003 (3)	0.003 (3)
C22	0.020 (3)	0.015 (3)	0.023 (3)	0.001 (2)	0.007 (2)	0.004 (2)
C23	0.024 (4)	0.028 (3)	0.027 (3)	−0.004 (3)	0.003 (3)	0.003 (3)
C24	0.038 (4)	0.023 (3)	0.032 (4)	−0.001 (3)	0.002 (3)	0.012 (3)
C25	0.041 (4)	0.029 (3)	0.022 (3)	0.006 (3)	0.003 (3)	0.010 (3)
C26	0.029 (4)	0.038 (4)	0.031 (4)	0.007 (3)	0.004 (3)	0.008 (3)
C27	0.024 (3)	0.020 (3)	0.025 (3)	0.003 (3)	0.007 (3)	0.007 (3)
C28	0.024 (3)	0.022 (3)	0.013 (3)	0.007 (3)	0.000 (2)	0.002 (2)
C29	0.024 (4)	0.023 (3)	0.036 (4)	−0.001 (3)	0.005 (3)	−0.011 (3)
C30	0.035 (4)	0.030 (4)	0.038 (4)	0.005 (3)	−0.004 (3)	−0.014 (3)
C31	0.047 (5)	0.033 (4)	0.030 (4)	0.013 (3)	0.003 (3)	−0.010 (3)
C32	0.050 (5)	0.033 (4)	0.031 (4)	0.015 (3)	0.019 (3)	−0.002 (3)
C33	0.029 (4)	0.030 (3)	0.027 (3)	0.010 (3)	0.006 (3)	−0.006 (3)
C34	0.016 (3)	0.021 (3)	0.023 (3)	0.003 (2)	0.005 (2)	0.000 (2)
C35	0.022 (3)	0.032 (3)	0.026 (3)	0.009 (3)	0.010 (3)	−0.003 (3)
C36	0.026 (4)	0.045 (4)	0.031 (4)	0.012 (3)	0.006 (3)	−0.004 (3)
C37	0.027 (4)	0.055 (5)	0.048 (5)	0.025 (4)	0.015 (3)	0.011 (4)
C38	0.022 (4)	0.046 (4)	0.027 (4)	0.017 (3)	0.001 (3)	0.011 (3)
C39	0.027 (4)	0.039 (4)	0.022 (3)	0.018 (3)	0.007 (3)	0.008 (3)

### Geometric parameters (Å, °)

**Table:**

Ru1—C1	1.903 (6)	C24—H24B	0.9900
Ru1—C3	1.915 (6)	C25—C26	1.518 (10)
Ru1—C2	1.919 (7)	C25—H25A	0.9900
Ru1—P1	2.3777 (15)	C25—H25B	0.9900
Ru1—P2	2.3780 (15)	C26—C27	1.532 (9)
P1—C16	1.849 (6)	C26—H26A	0.9900
P1—C4	1.877 (6)	C26—H26B	0.9900
P1—C10	1.878 (6)	C27—H27A	0.9900
P2—C34	1.862 (6)	C27—H27B	0.9900
P2—C28	1.869 (6)	C28—C33	1.527 (9)
P2—C22	1.873 (6)	C28—C29	1.536 (9)
O1—C1	1.155 (8)	C28—H28A	1.0000
O2—C2	1.160 (8)	C29—C30	1.523 (9)
O3—C3	1.161 (7)	C29—H29A	0.9900
C4—C5	1.529 (8)	C29—H29B	0.9900
C4—C9	1.537 (9)	C30—C31	1.523 (10)
C4—H4A	1.0000	C30—H30A	0.9900
C5—C6	1.543 (8)	C30—H30B	0.9900
C5—H5A	0.9900	C31—C32	1.493 (11)
C5—H5B	0.9900	C31—H31A	0.9900
C6—C7	1.507 (11)	C31—H31B	0.9900
C6—H6A	0.9900	C32—C33	1.533 (9)
C6—H6B	0.9900	C32—H32A	0.9900
C7—C8	1.538 (10)	C32—H32B	0.9900
C7—H7A	0.9900	C33—H33A	0.9900
C7—H7B	0.9900	C33—H33B	0.9900
C8—C9	1.523 (9)	C34—C35	1.535 (8)
C8—H8A	0.9900	C34—C39	1.538 (9)
C8—H8B	0.9900	C34—H34A	1.0000
C9—H9A	0.9900	C35—C36	1.536 (9)
C9—H9B	0.9900	C35—H35A	0.9900
C10—C11	1.538 (8)	C35—H35B	0.9900
C10—C15	1.544 (8)	C36—C37	1.517 (11)
C10—H10A	1.0000	C36—H36A	0.9900
C11—C12	1.535 (9)	C36—H36B	0.9900
C11—H11A	0.9900	C37—C38	1.543 (10)
C11—H11B	0.9900	C37—H37A	0.9900
C12—C13	1.510 (10)	C37—H37B	0.9900
C12—H12A	0.9900	C38—C39	1.522 (9)
C12—H12B	0.9900	C38—H38A	0.9900
C13—C14	1.512 (10)	C38—H38B	0.9900
C13—H13A	0.9900	C39—H39A	0.9900
C13—H13B	0.9900	C39—H39B	0.9900
C14—C15	1.540 (9)	C1TA—C7TA	1.310 (12)
C14—H14A	0.9900	C1TA—C2TA	1.3900
C14—H14B	0.9900	C1TA—C6TA	1.3900
C15—H15A	0.9900	C2TA—C3TA	1.3900
C15—H15B	0.9900	C2TA—H2TA	0.9500
C16—C21	1.534 (9)	C3TA—C4TA	1.3900
C16—C17	1.539 (8)	C3TA—H3TA	0.9500
C16—H16A	1.0000	C4TA—C5TA	1.3900
C17—C18	1.529 (8)	C4TA—H4TA	0.9500
C17—H17A	0.9900	C5TA—C6TA	1.3900
C17—H17B	0.9900	C5TA—H5TA	0.9500
C18—C19	1.531 (10)	C6TA—H6TA	0.9500
C18—H18A	0.9900	C7TA—H7TA	0.9800
C18—H18B	0.9900	C7TA—H7TB	0.9800
C19—C20	1.520 (10)	C7TA—H7TC	0.9800
C19—H19A	0.9900	C1TB—C7TB	1.338 (16)
C19—H19B	0.9900	C1TB—C2TB	1.3900
C20—C21	1.554 (9)	C1TB—C6TB	1.3900
C20—H20A	0.9900	C2TB—C3TB	1.3900
C20—H20B	0.9900	C2TB—H2TB	0.9500
C21—H21A	0.9900	C3TB—C4TB	1.3900
C21—H21B	0.9900	C3TB—H3TB	0.9500
C22—C27	1.543 (8)	C4TB—C5TB	1.3900
C22—C23	1.549 (8)	C4TB—H4TB	0.9500
C22—H22A	1.0000	C5TB—C6TB	1.3900
C23—C24	1.545 (9)	C5TB—H5TB	0.9500
C23—H23A	0.9900	C6TB—H6TB	0.9500
C23—H23B	0.9900	C7TB—H7TD	0.9800
C24—C25	1.512 (9)	C7TB—H7TE	0.9800
C24—H24A	0.9900	C7TB—H7TF	0.9800
			
C1—Ru1—C3	109.3 (3)	C25—C24—C23	111.1 (5)
C1—Ru1—C2	123.2 (3)	C25—C24—H24A	109.4
C3—Ru1—C2	127.5 (3)	C23—C24—H24A	109.4
C1—Ru1—P1	89.69 (19)	C25—C24—H24B	109.4
C3—Ru1—P1	92.00 (18)	C23—C24—H24B	109.4
C2—Ru1—P1	88.51 (18)	H24A—C24—H24B	108.0
C1—Ru1—P2	89.56 (19)	C24—C25—C26	110.6 (6)
C3—Ru1—P2	91.75 (18)	C24—C25—H25A	109.5
C2—Ru1—P2	88.80 (18)	C26—C25—H25A	109.5
P1—Ru1—P2	176.22 (5)	C24—C25—H25B	109.5
C16—P1—C4	102.8 (3)	C26—C25—H25B	109.5
C16—P1—C10	103.9 (3)	H25A—C25—H25B	108.1
C4—P1—C10	104.3 (3)	C25—C26—C27	111.8 (6)
C16—P1—Ru1	117.59 (19)	C25—C26—H26A	109.3
C4—P1—Ru1	111.03 (19)	C27—C26—H26A	109.3
C10—P1—Ru1	115.62 (19)	C25—C26—H26B	109.3
C34—P2—C28	102.7 (3)	C27—C26—H26B	109.3
C34—P2—C22	104.2 (3)	H26A—C26—H26B	107.9
C28—P2—C22	104.7 (3)	C26—C27—C22	111.4 (5)
C34—P2—Ru1	117.4 (2)	C26—C27—H27A	109.4
C28—P2—Ru1	110.9 (2)	C22—C27—H27A	109.4
C22—P2—Ru1	115.46 (19)	C26—C27—H27B	109.4
O1—C1—Ru1	177.3 (6)	C22—C27—H27B	109.4
O2—C2—Ru1	177.1 (6)	H27A—C27—H27B	108.0
O3—C3—Ru1	174.3 (6)	C33—C28—C29	109.2 (5)
C5—C4—C9	110.1 (5)	C33—C28—P2	113.0 (4)
C5—C4—P1	112.2 (4)	C29—C28—P2	116.6 (4)
C9—C4—P1	116.4 (4)	C33—C28—H28A	105.7
C5—C4—H4A	105.8	C29—C28—H28A	105.7
C9—C4—H4A	105.8	P2—C28—H28A	105.7
P1—C4—H4A	105.8	C30—C29—C28	111.3 (6)
C4—C5—C6	111.9 (5)	C30—C29—H29A	109.4
C4—C5—H5A	109.2	C28—C29—H29A	109.4
C6—C5—H5A	109.2	C30—C29—H29B	109.4
C4—C5—H5B	109.2	C28—C29—H29B	109.4
C6—C5—H5B	109.2	H29A—C29—H29B	108.0
H5A—C5—H5B	107.9	C29—C30—C31	111.1 (6)
C7—C6—C5	111.4 (6)	C29—C30—H30A	109.4
C7—C6—H6A	109.4	C31—C30—H30A	109.4
C5—C6—H6A	109.4	C29—C30—H30B	109.4
C7—C6—H6B	109.4	C31—C30—H30B	109.4
C5—C6—H6B	109.4	H30A—C30—H30B	108.0
H6A—C6—H6B	108.0	C32—C31—C30	110.0 (6)
C6—C7—C8	110.5 (6)	C32—C31—H31A	109.7
C6—C7—H7A	109.5	C30—C31—H31A	109.7
C8—C7—H7A	109.5	C32—C31—H31B	109.7
C6—C7—H7B	109.5	C30—C31—H31B	109.7
C8—C7—H7B	109.5	H31A—C31—H31B	108.2
H7A—C7—H7B	108.1	C31—C32—C33	112.4 (6)
C9—C8—C7	110.0 (6)	C31—C32—H32A	109.1
C9—C8—H8A	109.7	C33—C32—H32A	109.1
C7—C8—H8A	109.7	C31—C32—H32B	109.1
C9—C8—H8B	109.7	C33—C32—H32B	109.1
C7—C8—H8B	109.7	H32A—C32—H32B	107.9
H8A—C8—H8B	108.2	C28—C33—C32	111.8 (6)
C8—C9—C4	111.9 (6)	C28—C33—H33A	109.3
C8—C9—H9A	109.2	C32—C33—H33A	109.3
C4—C9—H9A	109.2	C28—C33—H33B	109.3
C8—C9—H9B	109.2	C32—C33—H33B	109.3
C4—C9—H9B	109.2	H33A—C33—H33B	107.9
H9A—C9—H9B	107.9	C35—C34—C39	109.6 (5)
C11—C10—C15	108.9 (5)	C35—C34—P2	112.6 (4)
C11—C10—P1	114.9 (4)	C39—C34—P2	113.5 (4)
C15—C10—P1	115.1 (4)	C35—C34—H34A	106.9
C11—C10—H10A	105.7	C39—C34—H34A	106.9
C15—C10—H10A	105.7	P2—C34—H34A	106.9
P1—C10—H10A	105.7	C34—C35—C36	110.1 (5)
C12—C11—C10	110.1 (5)	C34—C35—H35A	109.6
C12—C11—H11A	109.6	C36—C35—H35A	109.6
C10—C11—H11A	109.6	C34—C35—H35B	109.6
C12—C11—H11B	109.6	C36—C35—H35B	109.6
C10—C11—H11B	109.6	H35A—C35—H35B	108.2
H11A—C11—H11B	108.2	C37—C36—C35	111.1 (6)
C13—C12—C11	113.0 (6)	C37—C36—H36A	109.4
C13—C12—H12A	109.0	C35—C36—H36A	109.4
C11—C12—H12A	109.0	C37—C36—H36B	109.4
C13—C12—H12B	109.0	C35—C36—H36B	109.4
C11—C12—H12B	109.0	H36A—C36—H36B	108.0
H12A—C12—H12B	107.8	C36—C37—C38	111.7 (6)
C12—C13—C14	110.1 (6)	C36—C37—H37A	109.3
C12—C13—H13A	109.6	C38—C37—H37A	109.3
C14—C13—H13A	109.6	C36—C37—H37B	109.3
C12—C13—H13B	109.6	C38—C37—H37B	109.3
C14—C13—H13B	109.6	H37A—C37—H37B	107.9
H13A—C13—H13B	108.2	C39—C38—C37	112.3 (6)
C13—C14—C15	111.6 (5)	C39—C38—H38A	109.1
C13—C14—H14A	109.3	C37—C38—H38A	109.1
C15—C14—H14A	109.3	C39—C38—H38B	109.1
C13—C14—H14B	109.3	C37—C38—H38B	109.1
C15—C14—H14B	109.3	H38A—C38—H38B	107.9
H14A—C14—H14B	108.0	C38—C39—C34	109.7 (5)
C14—C15—C10	110.1 (5)	C38—C39—H39A	109.7
C14—C15—H15A	109.6	C34—C39—H39A	109.7
C10—C15—H15A	109.6	C38—C39—H39B	109.7
C14—C15—H15B	109.6	C34—C39—H39B	109.7
C10—C15—H15B	109.6	H39A—C39—H39B	108.2
H15A—C15—H15B	108.2	C7TA—C1TA—C2TA	120.6 (7)
C21—C16—C17	108.8 (5)	C7TA—C1TA—C6TA	119.3 (7)
C21—C16—P1	114.6 (4)	C2TA—C1TA—C6TA	120.0
C17—C16—P1	112.8 (4)	C3TA—C2TA—C1TA	120.0
C21—C16—H16A	106.7	C3TA—C2TA—H2TA	120.0
C17—C16—H16A	106.7	C1TA—C2TA—H2TA	120.0
P1—C16—H16A	106.7	C2TA—C3TA—C4TA	120.0
C18—C17—C16	110.5 (5)	C2TA—C3TA—H3TA	120.0
C18—C17—H17A	109.5	C4TA—C3TA—H3TA	120.0
C16—C17—H17A	109.5	C3TA—C4TA—C5TA	120.0
C18—C17—H17B	109.5	C3TA—C4TA—H4TA	120.0
C16—C17—H17B	109.5	C5TA—C4TA—H4TA	120.0
H17A—C17—H17B	108.1	C4TA—C5TA—C6TA	120.0
C17—C18—C19	110.6 (6)	C4TA—C5TA—H5TA	120.0
C17—C18—H18A	109.5	C6TA—C5TA—H5TA	120.0
C19—C18—H18A	109.5	C5TA—C6TA—C1TA	120.0
C17—C18—H18B	109.5	C5TA—C6TA—H6TA	120.0
C19—C18—H18B	109.5	C1TA—C6TA—H6TA	120.0
H18A—C18—H18B	108.1	C1TA—C7TA—H7TA	109.5
C20—C19—C18	112.4 (6)	C1TA—C7TA—H7TB	109.5
C20—C19—H19A	109.1	H7TA—C7TA—H7TB	109.5
C18—C19—H19A	109.1	C1TA—C7TA—H7TC	109.5
C20—C19—H19B	109.1	H7TA—C7TA—H7TC	109.5
C18—C19—H19B	109.1	H7TB—C7TA—H7TC	109.5
H19A—C19—H19B	107.9	C7TB—C1TB—C2TB	118.5 (8)
C19—C20—C21	112.6 (6)	C7TB—C1TB—C6TB	121.5 (8)
C19—C20—H20A	109.1	C2TB—C1TB—C6TB	120.0
C21—C20—H20A	109.1	C1TB—C2TB—C3TB	120.0
C19—C20—H20B	109.1	C1TB—C2TB—H2TB	120.0
C21—C20—H20B	109.1	C3TB—C2TB—H2TB	120.0
H20A—C20—H20B	107.8	C4TB—C3TB—C2TB	120.0
C16—C21—C20	109.2 (5)	C4TB—C3TB—H3TB	120.0
C16—C21—H21A	109.8	C2TB—C3TB—H3TB	120.0
C20—C21—H21A	109.8	C3TB—C4TB—C5TB	120.0
C16—C21—H21B	109.8	C3TB—C4TB—H4TB	120.0
C20—C21—H21B	109.8	C5TB—C4TB—H4TB	120.0
H21A—C21—H21B	108.3	C4TB—C5TB—C6TB	120.0
C27—C22—C23	108.6 (5)	C4TB—C5TB—H5TB	120.0
C27—C22—P2	114.9 (4)	C6TB—C5TB—H5TB	120.0
C23—C22—P2	114.9 (4)	C5TB—C6TB—C1TB	120.0
C27—C22—H22A	105.9	C5TB—C6TB—H6TB	120.0
C23—C22—H22A	105.9	C1TB—C6TB—H6TB	120.0
P2—C22—H22A	105.9	C1TB—C7TB—H7TD	109.5
C24—C23—C22	110.1 (6)	C1TB—C7TB—H7TE	109.5
C24—C23—H23A	109.6	H7TD—C7TB—H7TE	109.5
C22—C23—H23A	109.6	C1TB—C7TB—H7TF	109.5
C24—C23—H23B	109.6	H7TD—C7TB—H7TF	109.5
C22—C23—H23B	109.6	H7TE—C7TB—H7TF	109.5
H23A—C23—H23B	108.2		
			
C1—Ru1—P1—C16	166.5 (3)	C10—P1—C16—C17	68.9 (5)
C3—Ru1—P1—C16	57.2 (3)	Ru1—P1—C16—C17	−60.3 (5)
C2—Ru1—P1—C16	−70.3 (3)	C21—C16—C17—C18	62.4 (7)
P2—Ru1—P1—C16	−115.0 (9)	P1—C16—C17—C18	−169.4 (4)
C1—Ru1—P1—C4	−75.5 (3)	C16—C17—C18—C19	−57.9 (7)
C3—Ru1—P1—C4	175.2 (3)	C17—C18—C19—C20	52.4 (8)
C2—Ru1—P1—C4	47.7 (3)	C18—C19—C20—C21	−51.7 (9)
P2—Ru1—P1—C4	3.0 (10)	C17—C16—C21—C20	−59.8 (7)
C1—Ru1—P1—C10	43.0 (3)	P1—C16—C21—C20	173.0 (5)
C3—Ru1—P1—C10	−66.3 (3)	C19—C20—C21—C16	55.4 (8)
C2—Ru1—P1—C10	166.3 (3)	C34—P2—C22—C27	99.1 (5)
P2—Ru1—P1—C10	121.6 (9)	C28—P2—C22—C27	−153.4 (4)
C1—Ru1—P2—C34	−167.3 (3)	Ru1—P2—C22—C27	−31.1 (5)
C3—Ru1—P2—C34	−58.0 (3)	C34—P2—C22—C23	−28.0 (5)
C2—Ru1—P2—C34	69.5 (3)	C28—P2—C22—C23	79.6 (5)
P1—Ru1—P2—C34	114.1 (9)	Ru1—P2—C22—C23	−158.2 (4)
C1—Ru1—P2—C28	75.1 (3)	C27—C22—C23—C24	57.9 (7)
C3—Ru1—P2—C28	−175.6 (3)	P2—C22—C23—C24	−172.0 (4)
C2—Ru1—P2—C28	−48.1 (3)	C22—C23—C24—C25	−59.0 (8)
P1—Ru1—P2—C28	−3.5 (10)	C23—C24—C25—C26	57.0 (8)
C1—Ru1—P2—C22	−43.7 (3)	C24—C25—C26—C27	−55.6 (8)
C3—Ru1—P2—C22	65.6 (3)	C25—C26—C27—C22	56.3 (7)
C2—Ru1—P2—C22	−167.0 (3)	C23—C22—C27—C26	−56.9 (7)
P1—Ru1—P2—C22	−122.3 (9)	P2—C22—C27—C26	173.0 (4)
C3—Ru1—C1—O1	−7(12)	C34—P2—C28—C33	176.4 (5)
C2—Ru1—C1—O1	173 (12)	C22—P2—C28—C33	67.8 (5)
P1—Ru1—C1—O1	−99 (12)	Ru1—P2—C28—C33	−57.4 (5)
P2—Ru1—C1—O1	84 (12)	C34—P2—C28—C29	48.7 (5)
C1—Ru1—C2—O2	18 (11)	C22—P2—C28—C29	−59.9 (5)
C3—Ru1—C2—O2	−162 (11)	Ru1—P2—C28—C29	174.9 (4)
P1—Ru1—C2—O2	−70 (11)	C33—C28—C29—C30	56.1 (7)
P2—Ru1—C2—O2	107 (11)	P2—C28—C29—C30	−174.4 (5)
C1—Ru1—C3—O3	6(5)	C28—C29—C30—C31	−58.2 (8)
C2—Ru1—C3—O3	−174 (5)	C29—C30—C31—C32	57.0 (9)
P1—Ru1—C3—O3	96 (5)	C30—C31—C32—C33	−55.6 (9)
P2—Ru1—C3—O3	−85 (5)	C29—C28—C33—C32	−54.3 (7)
C16—P1—C4—C5	−177.6 (4)	P2—C28—C33—C32	174.3 (5)
C10—P1—C4—C5	−69.4 (5)	C31—C32—C33—C28	55.6 (8)
Ru1—P1—C4—C5	55.8 (5)	C28—P2—C34—C35	−178.1 (4)
C16—P1—C4—C9	−49.5 (5)	C22—P2—C34—C35	−69.1 (5)
C10—P1—C4—C9	58.7 (5)	Ru1—P2—C34—C35	60.0 (5)
Ru1—P1—C4—C9	−176.1 (4)	C28—P2—C34—C39	56.6 (5)
C9—C4—C5—C6	53.4 (7)	C22—P2—C34—C39	165.6 (4)
P1—C4—C5—C6	−175.2 (5)	Ru1—P2—C34—C39	−65.3 (5)
C4—C5—C6—C7	−55.3 (8)	C39—C34—C35—C36	−60.5 (7)
C5—C6—C7—C8	56.8 (8)	P2—C34—C35—C36	172.2 (5)
C6—C7—C8—C9	−58.1 (9)	C34—C35—C36—C37	57.5 (8)
C7—C8—C9—C4	57.7 (8)	C35—C36—C37—C38	−53.2 (8)
C5—C4—C9—C8	−55.5 (8)	C36—C37—C38—C39	53.0 (8)
P1—C4—C9—C8	175.4 (5)	C37—C38—C39—C34	−55.7 (8)
C16—P1—C10—C11	−100.3 (4)	C35—C34—C39—C38	59.4 (7)
C4—P1—C10—C11	152.2 (4)	P2—C34—C39—C38	−173.8 (4)
Ru1—P1—C10—C11	30.0 (5)	C7TA—C1TA—C2TA—C3TA	−178.5 (9)
C16—P1—C10—C15	27.4 (5)	C6TA—C1TA—C2TA—C3TA	0.0
C4—P1—C10—C15	−80.0 (5)	C1TA—C2TA—C3TA—C4TA	0.0
Ru1—P1—C10—C15	157.8 (4)	C2TA—C3TA—C4TA—C5TA	0.0
C15—C10—C11—C12	57.3 (6)	C3TA—C4TA—C5TA—C6TA	0.0
P1—C10—C11—C12	−171.9 (4)	C4TA—C5TA—C6TA—C1TA	0.0
C10—C11—C12—C13	−56.8 (7)	C7TA—C1TA—C6TA—C5TA	178.5 (9)
C11—C12—C13—C14	55.3 (7)	C2TA—C1TA—C6TA—C5TA	0.0
C12—C13—C14—C15	−55.8 (7)	C7TB—C1TB—C2TB—C3TB	179.6 (11)
C13—C14—C15—C10	58.6 (7)	C6TB—C1TB—C2TB—C3TB	0.0
C11—C10—C15—C14	−58.6 (7)	C1TB—C2TB—C3TB—C4TB	0.0
P1—C10—C15—C14	170.7 (4)	C2TB—C3TB—C4TB—C5TB	0.0
C4—P1—C16—C21	−57.4 (5)	C3TB—C4TB—C5TB—C6TB	0.0
C10—P1—C16—C21	−166.0 (5)	C4TB—C5TB—C6TB—C1TB	0.0
Ru1—P1—C16—C21	64.9 (5)	C7TB—C1TB—C6TB—C5TB	−179.6 (11)
C4—P1—C16—C17	177.4 (4)	C2TB—C1TB—C6TB—C5TB	0.0
